# Feedback reports to the general practitioner (GP) on the patients’ experiences: are GPs interested, and is this interest associated with GP factors and patient experience scores?

**DOI:** 10.1093/fampra/cmad019

**Published:** 2023-02-28

**Authors:** Øyvind A Bjertnæs, Rebecka M Norman, Torunn B Eide, Olaf Holmboe, Hilde H Iversen, Kjetil Telle, Jose M Valderas

**Affiliations:** Department of Health Services Research, Division for Health Services, Norwegian Institute of Public Health, Oslo 0473, Norway; Department of Health Services Research, Division for Health Services, Norwegian Institute of Public Health, Oslo 0473, Norway; Department of General Practice, University of Oslo, Oslo, Norway; Department of Health Services Research, Division for Health Services, Norwegian Institute of Public Health, Oslo 0473, Norway; Department of Health Services Research, Division for Health Services, Norwegian Institute of Public Health, Oslo 0473, Norway; Department of Health Services Research, Division for Health Services, Norwegian Institute of Public Health, Oslo 0473, Norway; Department of Family Medicine, National University Health System, Level 9, Singapore, Singapore

**Keywords:** general practice, general practitioner, health care surveys, patient satisfaction, physician–patient relations, quality improvement

## Abstract

**Background:**

Patient experience feedback is key in patient centred health systems, but empirical evidence of general practitioner (GP) interest in it is sparse. We aimed to: (i) quantitatively estimate the level of GP interest for feedback reports on patient experience; (ii) explore determinants of such interest; and (iii) examine potential association between a priori interest and patient experience.

**Methods:**

The patient experience survey included maximum 300 randomly selected patients for each of 50 randomly selected GPs (response rate 41.4%, *n* = 5,623). GPs were sent a postal letter offering feedback reports and were grouped according to their replies: (i) interested in the report; (ii) not interested. Associations between interest and GP variables were assessed with Chi-square tests and multivariate logistic regression, while associations between interest and scores for 5 patient experiences scales were assessed with multilevel regression models.

**Results:**

About half (*n* = 21; 45.7%) of the GPs showed interest in the report by asking to receive the report. The only GP variable associated with a priori interest was being a specialist in general practice (58.6% vs. 23.5% for those without) (*P* = 0.021). Interest was significantly associated with the practice patient experience scale (4.1 higher score compared with those not interested, *P* = 0.048). Interest in the report had small and nonsignificant associations with the remaining patient experience scales.

**Conclusions:**

Almost half of the GPs, and almost 3 in 5 of specialists in general practice, were interested in receiving a GP-specific feedback report on patient experiences. Interest in the report was generally not related to patient experience scores.

**Key messages**

- Empirical evidence of GP interest in patient experience feedback is sparse.
- About half of the GPs were interested in receiving a feedback report.
- Specialists in general practice were more interested than nonspecialists.
- Interest was generally not related to patient experience scores.

## Background

Patient centredness is a core part of health care quality^1,2^ which is often measured with patient experience surveys. Several large-scale initiatives exist for primary care or general practice, including the General Practitioner (GP) Patient Survey in United Kingdom^3^ and the Consumer Assessment of Healthcare Providers and Systems (CAHPS) Clinician and Group Survey in the United States.^4^ While many scientific studies have been published on instruments, data collection procedures and analytical approaches in these and related initiatives, a review of the literature evidenced less attention to the actual use of these data by primary health care practices.^5^ Failing to effectively close the feedback loop is a clear limitation given that a main goal of quality measurement is to facilitate quality improvement.

Current research confirms both a general positive attitude to patient feedback in primary health care practices, and varied and often negative views (especially GPs in United Kingdom) on the utility, validity, and reliability of actual patient experience surveys.^5–11^ One important and frequent limitation arises when the feedback reports are presented at aggregate levels above the individual GP level, especially the practice level.^6,9,11^ While results for the practice or higher levels are useful reference points for individual GP results, previous research documents substantial variation in patient experiences between individual primary care physicians,^12^ even within the same practice.^9,13^ Thus, practice-level reports can mask differences between GPs within the same practice and make results less relevant and useful for individual GPs, thereby limiting the possibility to inform quality improvement. Only a few studies have studied doctors’ engagement with surveys at the individual doctor level.^7,8,14^ These studies showed engagement and personal commitment by doctors, but also scepticism as to the credibility, reliability, and validity of findings.^7,8,14^ The studies had questionable generalizability, which combined with the small amount of studies evidences the need for more research on GP engagement with surveys conducted at the GP level.^5,6,9–11^

The main objectives of this study were to: (i) quantitatively estimate the level of interest for individual GP reports on patient experiences among GPs; (ii) explore determinants of interest for the report, including GP factors like gender and age; and (iii) examine potential association between a priori interest for the report and patient experiences scores.

## Methods

### Setting

All residents in Norway are entitled to a regular GP, and around 99% of the population are part of the regular GP scheme.^15^ Norwegian GPs are gatekeepers for the national insurance scheme, and patients are referred from a GP to specialized medical care when needed. The GP practices are, in general, small units with 2–5 GPs, most of which are self-employed.^16^ Normally, there are 1 or more receptionists as well as staff for sampling and analysing point-of-care tests at the GP practice. Since 2017, GPs entering the regular GP scheme in Norway are required to be specialists in general medicine or under specialist training. There are exceptions for GPs that entered the scheme before 2017 and locum doctors.

### Participants

The Norwegian Institute of Public Health (NIPH) conducted a national cross-sectional GP patient experience survey starting in September 2021, with 10 patients randomly selected (simple random sampling) from each of a stratified, random sample of 2,000 GPs (*N* = 20,000). The response rate in the national survey was 41.9%. The national results showed that many patients have positive experiences with their GPs and GP practices on a number of areas, but also identified clear improvement areas including accessibility and waiting times. To obtain robust estimates at the GP level, we first selected 50 GPs from the main sample, using simple random sampling. The probability of ending up with GPs from the same practice was low, but we made no restrictions on this. We then randomly selected 290 additional patients from each of these GPs, or all patients if the number of remaining patients on the list was below 290. The 50 selected GPs were not informed in advance about addressing their patients and thus could not facilitate patient participation. All inhabitants in Norway are entitled to an individual regular GP, as part of the national GP scheme. Patients were instructed to evaluate their current regular GP, but also informed that they could evaluate another GP if they had more contact with another GP. Three of 50 GPs resigned from the GP practice between sample construction (June 2021) and survey start (September 2021) and were excluded from the study. The inclusion criteria were patients aged 16 years and older with minimum 1 contact with the GP the last 12 months. Patients registered in a national digital portal received a digital invitation to the survey with an electronic response option, while the others were mailed a postal invitation letter with an electronic response option. Two reminders were sent to nonrespondents, both including a pen-and-paper questionnaire and electronic response option. Data collection was completed in January 2022. The response rate in the GP-level subsample was 41.4% (*n* = 5,623), with patient response rates for the 47 GPs ranging from 20.3% to 58.5%.

### Measurements

The generic patient experience questionnaire for GPs (PEQ-GP) consists of 5 scales with 18 items^17^: assessment of GP (8 items), cooperation (2 items), patient enablement (3 items), accessibility (2 items), and practice (3 items). All items had a 5-point response format ranging from 1 (not at all) to 5 (to a very large extent). Three of the scales include items with direct assessments of the GP, namely patient centredness, patient enablement (effect of GP on coping and understanding of health conditions), and cooperation (coordination and cooperation with other services). Accessibility relates to waiting times for appointments (acute, ordinary), while the practice scale relates to assessments of the organization of the GP practice, reception and auxiliary staff.^17^ The scales were linearly transformed to 0–100, where 100 is the best possible score. An English version of the questionnaire can be found in Appendix 1.

GP variables were collected from the national GP registry, including age, gender, number of years as GP in general and as restricted to the same practice, specialty status in general practice, number of GPs in practice, patient list size, available spots on patient list (full patient list, or not full and the number of available spots), and employment status (self-employed vs. fixed salary from the municipality). Number of years as GP and in the same practice lacked detailed information above 19 years (coded 20 if years were >19).

We produced a GP-level feedback report for each GP in the subsample (*n* = 47), which included tables with their own results (%, mean) for all questions/indicators and results for the subsample as reference points. We wanted to measure individual GPs’ interest in the feedback report. The traditional approach would be to send a letter to the GP using the GP practice address, but this would in many cases involve practice staff and possibly distort the intervention. Furthermore, we wanted to avoid methods creating social desirability bias, like telephone or face-to-face approaches. We gathered interest in receiving the data by offering individual feedback reports to all 47 GPs, via a letter which was sent to each GPs home address. The letter was sent to the GPs 9–10th of June 2022, shortly after NIPH published national results from the main survey.^18^ The letter briefly informed that the NIPH had conducted a patient experience survey and offered them a report with results for their patients. GPs who wanted the report could reply to a project e-mail address or by SMS/telephone. The GPs were asked to reply before July 2022, and no reminders were sent. One letter was returned because of unknown address. This GP and the corresponding patient data were excluded from the analysis in this study.

### Statistical analysis

To assess representativeness, we compared the subsample (*n* = 47) with the rest of the total sample in the main survey (*n* = 1,900) on available GP variables, using Chi-square tests for categorical variables and 1-way ANOVA for continuous variables. The associations between interest in the report and GP variables were first assessed with Chi-square tests, with all predictors transformed to dichotomous variables. Secondly, we ran a multivariate logistic regression with interest in the report as dependent variable and all GP variables included as predictors. In the multivariate logistic regression, all continuous variables were included without transformation. The association between interest in the report and patient experiences scores was assessed by running multilevel regression models, with the scales and items as dependent variables, the GP level included as random intercept (*n* = 47), and interest in the report included as predictor at level 2 (GP level). In multilevel regression, the variation is separated at different levels, e.g. the patient and the GP level, and allows inclusion of variables at each level to explain the variance at that level.^19^ We also computed the intraclass correlation coefficient for each scale and item (the variation between GPs divided by the total variation), by running empty multilevel models with GPs (*n* = 47) as random intercept.

Based on previous literature,^5–11,14^ we expected a rather modest amount of GP interest in the feedback reports about patient experiences. We are not aware of previous similar studies, which means that our study was exploratory without a priori hypothesis in relation to other associations.

All analyses were conducted with SPSS28.0.

## Results

We found small differences between the GP-level subsample and the total sample in the main survey regarding GP background variables (Table 1), with the largest difference pertaining to the gender distribution: 68.1% of GPs in the subsample were men, compared with 55.2% in the total sample (*P* = 0.078). GPs working in a group practice (vs. solo) and GPs having no available spots on the GP list were also slightly overrepresented in the GP-level sample, but none of the differences reached statistical significance (*P* = 0.204 and *P* = 0.371, respectively).

**Table 1.** Representativeness of random sample of GPs in relation to the main survey.

	GP-level subsample (*n* = 47)	Rest of total sample (*n* = 1,900)	*P*
GP factors			
Men, *n* (%)	32 (68.1)	1,048 (55.2)	0.078
Mean age (SD)	47.8 (12.3)	48.4 (11.4)	0.693
Specialist in general practice, *n* (%)	30 (63.8)	1,225 (64.5)	0.927
Twenty years or more as GP, *n* (%)^a^	14 (29.8)	548 (28.8)	0.888
Twenty years or more as GP in the same practice, *n* (%)^a^	14 (29.8)	512 (26.9)	0.655
Practice factors			
Mean number of patients on the list (SD)	1,113.6 (376.8)	1,093.9 (371.6)	0.720
No available spots on patient list, *n* (%)	15 (31.9)	496 (26.1)	0.371
Group practice, *n* (%)	45 (95.7)	1,714 (90.2)	0.204
Mean number of GPs in practice (SD)	4.0 (1.8)	3.8 (2.0)	0.659
Fixed salary GP, *n* (%)	5 (10.6)	276 (14.5)	0.454

^a^Cut-off related to 20 years because we lack detailed data above 19 years.

45.7% (*n* = 21) of the GPs showed interest in the report by asking to receive the report from the NIPH (Table 2). Interest in the report varied significantly between GP specialists and those without specialty: 58.6% of the former asked for the report, while 23.5% of the latter asked for the report (*P* = 0.021). Of GPs with a group practice, 47.7% were interested in the report, while none of those in solo practice showed an interest. However, only 2 GPs in the subsample worked in a solo practice, so this large difference was not significant (*P* = 0.185). None of the other variables were associated with interest in the report in bivariate analysis (Table 2). A multivariate logistic regression with all GP variables as predictors confirmed that being a GP specialist had a significant effect on interest in the report (*B* = 2.241, *P* = 0.038), but none of the other variables had a significant effect (results not shown here).

**Table 2.** GP- and practice-level factors by interest in individual feedback report^a^.

	Asked for the report, *n* = 17 (45.7%)	Did not ask for the report, *n* = 25 (54.3%)	*P*
Gender			0.695
Men, *n* (%)	14 (43.8)	18 (56.2)	
Women, *n* (%)	7 (50.0)	7 (50.0)	
Age^b^			0.536
45 or below, *n* (%)	9 (40.9)	13 (59.1)	
Older than 45, *n* (%)	12 (50.0)	12 (50.0)	
Specialist in general practice			0.021
Yes, *n* (%)	17 (58.6)	12 (41.4)	
No, *n* (%)	4 (23.5)	13 (76.5)	
Years as GP			0.484
20 or more, *n* (%)	7 (53.8)	6 (46.2)	
Less than 20, *n* (%)	14 (42.4)	19 (57.6)	
Length of patient list			0.806
More than average, *n* (%)	10 (47.6)	11 (52.4)	
Less than average, *n* (%)	11 (44.0)	14 (56.0)	
Available spots on patient list			0.301
Yes, *n* (%)	13 (40.6)	19 (59.4)	
No, *n* (%)	8 (57.1)	6 (42.9)	
Group practice^c^			0.185
Yes, *n* (%)	21 (47.7)	23 (52.3)	
No, *n* (%)	0 (0.0)	2 (100.0)	
Fixed salary			0.788
Yes, *n* (%)	2 (40.0)	3 (60.0)	
No, *n* (%)	19 (46.3)	22 (53.7)	

^a^One GP had unknown address and is excluded here (*n* = 46).

^b^Cut-off based on age distribution of GPs (equal/less than 50% of GPs, the rest).

^c^Equals 1 vs. >1 GPs in practice.

In multilevel analysis with the GP level as random intercept (Table 3), the intraclass correlation coefficient ranged from 0.081 (cooperation scale) to 0.159 (accessibility scale). Showing an interest in the report was significantly associated with the practice scale: those with an interest had 4.1 higher score on the practice scale compared with those not interested (*P* = 0.048). The effect on accessibility was close to the effect of the practice scale (estimate: 3.488), but was not significant (*P* = 0.326). Interest in the report had small and nonsignificant associations with the remaining scales. Results for items followed the same pattern as for scales, with a significant association only found between interest in the report and the practice scale item “other employees helpful and competent” (*P* = 0.038).

**Table 3.** Multilevel regression models: association between GPs interest in individual feedback report and patient experience scores.

	ICC^a^	Interest in report^b^	
		Estimate	*P*
GP scale	0.131	−0.257	0.894
1 GP takes you seriously	0.101	−0.886	0.661
2 GP spends enough time with you	0.123	−0.510	0.847
3 GP talks to you in a way you understand	0.085	−0.533	0.752
4 GP is professionally competent	0.120	0.317	0.875
5 GP shows interest in your situation	0.111	−0.898	0.693
6 GP includes you as much as you would like in decisions concerning you	0.097	0.271	0.895
7 GP provide sufficient information about health problems and treatment	0.100	0.606	0.783
8 GP provide sufficient information about use/side effects of medication	0.064	0.486	0.827
Practice scale	0.137	4.100	0.048
1 GP practice well organized	0.162	5.095	0.057
2 Other employees helpful and competent	0.104	4.258	0.038
3 Treated with courtesy and respect at the reception	0.091	3.233	0.093
Accessibility scale	0.159	3.488	0.326
1 Waiting time for your last urgent appointment acceptable	0.107	4.477	0.178
2 Waiting time for appointments that are not urgent acceptable	0.184	3.092	0.447
Enablement scale	0.085	−0.138	0.947
1 Contact with GP make you better able to understand your health problems	0.080	−0.069	0.974
2 Contact with GP make you better able to cope with your health problems	0.076	0.014	0.995
3 Contact with GP better help you to stay healthy	0.066	−0.415	0.842
Cooperation scale	0.081	0.848	0.671
1 GP is good at coordinating the range of health services available to you	0.084	1.134	0.584
2 GP cooperates well with other services you need	0.074	0.684	0.739

^a^Intraclass correlation coefficient, i.e. the variation between GPs divided by the total variation.

^b^Separate model for each scale/item with GP as random intercept, and interest as predictor at GP level.

## Discussion

Almost half of the GPs, and almost 3 in 5 of specialists in general practice, were interested in receiving an individual feedback report on patient experiences. Interest in the report was not related to patient experience scores, except for the practice scale.

In contrast to the Quality and Outcomes Framework (QOF) in United Kingdom, linking performance with payments and with professional concerns from many providers,^20^ no financial or other strings were attached to the GP-level results. As such, our study provided an opportunity to study an aspect of patient centredness among GPs, i.e. interest in feedback reports about their patients’ experiences. This low-stake approach taps into values of universalism and benevolence, which previous research has found missing in the QOF in United Kingdom.^20^ Obviously, obtaining the report is also a prerequisite for having the possibility to use the results for practice evaluation and improvement, which could be seen as a first necessary step for GP activation for quality improvement. The Commonwealth Fund’s international health policy survey has documented large variations between countries regarding the collection and use of patient experience data among primary care doctors^21^: only 4% of Norwegian and French doctors reported quarterly/yearly use, compared with 81% in the United Kingdom. The survey also asked about the use of other performance measures like clinical results, patient-reported outcome measures and patients’ use of hospital services and primary care-out-of-hours services, and also for these measures Norwegian GPs reported less use than many of the other countries.^21^ Based on the low level of use of patient experience data among Norwegian GPs and the scepticism found in previous international studies,^7,8,14^ we expected a rather modest amount of interest in the report, and lower than what was actually found. We have 2 explanations for this positive finding. First, conducting a patient experience survey and analysis require infrastructure and technical competence in survey methodology and statistics, which are barriers for collection and use of this kind of data.^22^ In our study, and in countries with national survey systems like the United Kingdom, this barrier is removed because professional vendors are conducting the survey, analysis, and reporting. Second, no strings were attached to the feedback report (i.e. no incentives, templates, or process requirements) and results were only fed back to the individual GP. An important goal with the subsample was to underpin internal quality evaluation and improvement, not external benchmarking. Thus, the potential negative consequences of poor results for GPs were very low or nonexistent.

Even though the GPs only had to ask for the report by SMS/e-mail or telephone, more than half of the doctors did not. It seems like other barriers are at work, including organizational and professional barriers, e.g. time pressure, competing priorities, and scepticism to patient feedback.^22^ A recent Norwegian study found that GP-specific feedback reports about antibiotic use were very well received, but unlike our reports these were part of an educational programme and gave credits in continuing medical education.^23^ In our study, none of the background factors about GPs were related to interest in the report, apart from specialists being much more interested. In Norway, a competence regulation demands that all GPs entering the regular GP scheme after 1st of March 2017 are specialists in general practice or under training to be specialists. The goal is to increase the percentage of specialists over time. Based on the findings from our study, this might also have a positive effect on the interest in feedback reports about patient experiences. However, raising their interest in feedback reports is not the same as convincing them of the validity and value of results and using them to evaluate and improve practice, as previous research has clearly documented.^5–11,14^ Our study meets some of the challenges described in the literature: we provided individual reports for each GP, not aggregated to practice- or higher-level reports,^7,9^ and the initiative was conducted in a low-stake context suitable for internal evaluation and improvement. We believe that these factors increase the probability of use of data for internal evaluation and improvement, but also that more development and research are needed. We propose the following initiatives and research to secure stronger uptake and impact of the patient experience survey in the coming years: (i) including analysis of patient comments in feedback reports to GPs to increase relevance and actionability,^9^ by developing and validating machine learning for automatic sentiment analysis of free-text comments from patients. This is ongoing work in Norway using data from the GP patient experience survey, financed by the Norwegian Research Council^24^; (ii) larger studies to test and implement continuous, electronic surveys with real-time feedback and time series.^25^ This approach will be piloted in the GP patient experience survey in 2023; and (iii) stronger involvement of GPs to secure relevance, usefulness, and impact, including surveys and qualitative interviews about attitudes, usefulness and the content of feedback reports (part of NRC project), and the inclusion of data/reports in structured quality improvement programmes offered to GPs in existing structures giving credits in specialist training and continuing medical education. As part of this it is important to establish how many GPs actually wish to receive direct patient feedback for improving their consultations and practice organization, which is fundamental for actually acting upon this feedback.

Interest in the report was not related to patient experience scores, except for the practice scale. Thus, we found little evidence of selection bias, meaning that both high and low performing GPs asked for the report. Previous research shows that the impact of patient experience surveys is strongest for low performers,^26^ implying that it is important with variation in GP performance among participants to achieve impact and improvements. Naturally, a range of other individual and organizational factors are important to create changes,^27^ but the low degree of selection bias was positive both from a methodological and quality improvement perspective.

### Limitations

The subsample of GPs had similar characteristics as the main sample. However, the low number of GPs in the study was a limitation, negatively influencing the power to detect all existing associations. The reason for including only 50 GPs was budget restrictions, especially large costs related to printing and postage for the subsample and the main sample. Many associations were weak, but some were close to significant and should be studied with larger GP samples, e.g. the association between practice size and interest in the report, and between interest and patient-perceived accessibility. Thus, further research with larger GP samples is warranted, following proper sample size calculations. Another limitation is external validity, which is restricted to countries with similar trajectories regarding measurements of patient experiences. Following findings in the Commonwealth Fund international health policy survey among 11 western countries,^21^ countries like France, Germany, Canada, and Switzerland resembles Norway with little experience with patient experience surveys among primary care doctors, while our study is probably less generalizable to countries with more experience like United Kingdom and United States.

## Conclusions

Almost half of the GPs, and almost 3 in 5 of specialists in general practice, were interested in receiving an individual feedback report on patient experiences. Interest in the report was not related to patient experience scores, except for the practice scale. It seems like feedback reports fills a knowledge gap and reach both high and low performing GPs. These are both important factors to facilitate and motivate for quality improvement in general practice. Further studies with larger GP samples and additional data from the GPs are warranted.

We recommend further initiatives and research to secure stronger uptake and impact of patient experience surveys, including analysis of patient comments in feedback reports to GPs, larger studies for testing and implementing continuous, electronic surveys with real-time feedback, and stronger involvement of GPs in these processes to secure relevance, usefulness, and impact.

## Supplementary Material

cmad019_suppl_Supplementary_Appendix_1

cmad019_suppl_Supplementary_STROBE_Checklist

## Data Availability

The dataset is available from the corresponding author on reasonable request.

## References

[1] Berwick DM , Nolan TW, Whittington J. The triple aim: care, health, and cost. Health Aff (Millwood). 2008;27(3):759–769. 18474969 10.1377/hlthaff.27.3.759

[2] Carinci F , Van Gool K, Mainz J, Veillard J, Pichora EC, Januel JM, Arispe I, Kim SM, Klazinga NS; OECD Health Care Quality Indicators Expert Group. Towards actionable international comparisons of health system performance: expert revision of the OECD framework and quality indicators. Int J Qual Health Care. 2015;27(2):137–146. 25758443 10.1093/intqhc/mzv004

[3] Roberts MJ , Campbell JL, Abel GA, Davey AF, Elmore NL, Maramba I, Carter M, Elliott MN, Roland MO, Burt JA. Understanding high and low patient experience scores in primary care: analysis of patients’ survey data for general practices and individual doctors. BMJ. 2014;349:g6034. 25389136 10.1136/bmj.g6034 PMC4230029

[4] Dyer N , Sorra JS, Smith SA, Cleary PD, Hays RD. Psychometric properties of the Consumer Assessment of Healthcare Providers and Systems (CAHPS®) clinician and group adult visit survey. Med Care. 2012;50(Suppl):S28–S34. 23064274 10.1097/MLR.0b013e31826cbc0d PMC3480671

[5] Baldie DJ , Guthrie B, Entwistle V, Kroll T. Exploring the impact and use of patients’ feedback about their care experiences in general practice settings—a realist synthesis. Fam Pract. 2018;35(1):13–21. 28985368 10.1093/fampra/cmx067 PMC6191909

[6] Asprey A , Campbell JL, Newbould J, Cohn S, Carter M, Davey A, Roland M. Challenges to the credibility of patient feedback in primary healthcare settings: a qualitative study. Br J Gen Pract. 2013;63(1):e200–e208. 23561787 10.3399/bjgp13X664252 PMC3582979

[7] Farrington C , Burt J, Boiko O, Campbell J, Roland M. Doctors’ engagements with patient experience surveys in primary and secondary care: a qualitative study. Health Expect. 2017;20(608):385–394. 27124310 10.1111/hex.12465 PMC5433536

[8] Boiko O , Campbell JL, Elmore N, Davey AF, Roland M, Burt J. The role of patient experience surveys in quality assurance and improvement: a focus group study in English general practice. Health Expect. 2015;18(6):1982–1994. 25366992 10.1111/hex.12298 PMC5810660

[9] Burt J , Campbell J, Abel G, Aboulghate A, Ahmed F, Asprey A, Barry H, Beckwith J, Benson J, Boiko O, et al. Improving patient experience in primary care: a multimethod programme of research on the measurement and improvement of patient experience. Southampton (UK): NIHR Journals Library; 2017. PMID: 28654227. 28654227

[10] Heje HN , Vedsted P, Olesen F. General practitioners’ experience and benefits from patient evaluations. BMC Fam Pract. 2011;12:116. doi:10.1186/1471-2296-12-116. PMID: 22040039; PMCID: PMC3217866. 22040039 PMC3217866

[11] Edwards A , Evans R, White P, Elwyn G. Experiencing patient-experience surveys: a qualitative study of the accounts of GPs. Br J Gen Pract. 2011;61(585):157–166. 21439173 10.3399/bjgp11X567072 PMC3063044

[12] Safran DG , Karp M, Coltin K, Chang H, Li A, Ogren J, Rogers WH. Measuring patients’ experiences with individual primary care physicians. Results of a statewide demonstration project. J Gen Intern Med. 2006;21(1):13–21. 16423118 10.1111/j.1525-1497.2005.00311.x PMC1484603

[13] Bjertnaes OA , Lyngstad I, Malterud K, Garratt A. The Norwegian EUROPEP questionnaire for patient evaluation of general practice: data quality, reliability and construct validity. Fam Pract. 2011;28(3):342–349. 21078822 10.1093/fampra/cmq098

[14] Hill JJ , Asprey A, Richards SH, Campbell JL. Multisource feedback questionnaires in appraisal and for revalidation: a qualitative study in UK general practice. Br J Gen Pract. 2012;62(598):e314–e321. 22546590 10.3399/bjgp12X641429 PMC3338052

[15] Saunes IS , Karanikolos M, Sagan AN. Health system review. Health Syst Transit. 2020;22(1):i-163. 32863241

[16] Eide TB , Straand J, Björkelund C, Kosunen E, Thorgeirsson O, Vedsted P, Rosvold EO. Differences in medical services in Nordic general practice: a comparative survey from the QUALICOPC study. Scand J Prim Health Care. 2017;3(2):1–10. doi:10.1080/02813432.2017.1358856. PMID: 28768442. 28768442

[17] Holmboe O , Iversen HH, Danielsen K, Bjertnaes O. The Norwegian patient experiences with GP questionnaire (PEQ-GP): reliability and construct validity following a national survey. BMJ Open. 2017;7(9):e016644. 10.1136/bmjopen-2017-016644 PMC5640105 28971964

[18] Norman RM , Bjertnæs ØA, Danielsen K, Holmboe O. O. Pasienterfaringer med fastlegen og fastlegekontoret i 2021/2022 [Patient experience with the general practitioner and the general practitioner office in 2021/2022]. PasOpp-rapport. Oslo: Folkehelseinstituttet; 2022. p. 566.

[19] Peugh JL. A practical guide to multilevel modeling. J Sch Psychol. 2010;48(1):85–112. 20006989 10.1016/j.jsp.2009.09.002

[20] Khan N , Rudoler D, McDiarmid M, Peckham S. A pay for performance scheme in primary care: meta-synthesis of qualitative studies on the provider experiences of the quality and outcomes framework in the UK. BMC Fam Pract. 2020;21(1):1–20. 32660427 10.1186/s12875-020-01208-8 PMC7359468

[21] Skudal KE , Holmboe O, Bjertnæs ØA, Telle K. Commonwealth Fund-undersøkelsen blant allmennleger i elleve land i 2019: tabellrapport [Commonwealth Fund survey of general practitioners in eleven countries in 2019: table report]. Oslo: Folkehelseinstituttet; 2019.

[22] Davies E , Cleary PD. Hearing the patient’s voice? Factors affecting the use of patient survey data in quality improvement. Qual Saf Health Care. 2005;14(6):428–432. 16326789 10.1136/qshc.2004.012955 PMC1744097

[23] Eide TB , Øyane N, Høye S. Promoters and inhibitors for quality improvement work in general practice: a qualitative analysis of 2715 free-text replies. BMJ Open Qual. 2022;11(4):e001880. 10.1136/bmjoq-2022-001880 PMC9557324 36207051

[24] Norwegian Research Council. Machine learning on free text comments [accessed 2022 Dec 7]. https://prosjektbanken.forskningsradet.no/project/FORISS/331770?Kilde=FORISS&distribution=Ar&chart=bar&calcType=funding&Sprak=no&sortBy=date&sortOrder=desc&resultCount=30&offset=90&LTP.1=LTP2+Fornyelse+i+offentlig+sektor%20

[25] Iversen HH , Haugum M, Bjertnaes O. Reliability and validity of the Psychiatric Inpatient Patient Experience Questionnaire—Continuous Electronic Measurement (PIPEQ-CEM). BMC Health Serv Res. 2022;22(1):1–13. 35821137 10.1186/s12913-022-08307-5 PMC9275271

[26] Haugum M , Danielsen K, Iversen HH, Bjertnaes O. The use of data from national and other large-scale user experience surveys in local quality work: a systematic review. Int J Qual Health Care. 2014;26(6):592–605. 25142685 10.1093/intqhc/mzu077

[27] Weiner BJ. A theory of organizational readiness for change. Implement Sci. 2009;4:1–9. 19840381 10.1186/1748-5908-4-67 PMC2770024

