# Supplementary material for: Feedback reports to the general practitioner (GP) on the patients’ experiences: are GPs interested, and is this interest associated with GP factors and patient experience scores?
Source: Fam Pract. 2023 Feb 28;40(5-6):682–8. doi: 10.1093/fampra/cmad019 (PMC10745253; doi:10.1093/fampra/cmad019)
Supplement: cmad019_suppl_Supplementary_Appendix_1 [file cmad019_suppl_supplementary_appendix_1.pdf]

FHI
Folkehelseinstituttet

Your experience of your GP and GP surgery

PasOpp Fastlege 2021

We ask that you evaluate your GP in this form.
If you have had more contact with another GP, you can consider that doctor instead.

The form is read by a machine. We ask you to use a blue or black pen and enter one X for each question.
Like this: ☒ *not like this:* ☐X

Availability

1. When were you last in touch with your GP/ GP surgery?

- ☐ Less than 1 month ago
- ☐ 1-3 months ago
- ☐ 4-6 months ago
- ☐ 7-12 months ago
- ☐ More than 12 months ago

2. Is it difficult to get in touch with your GP surgery via the telephone?

- ☐ Not at all
- ☐ To a small extent
- ☐ To some extent
- ☐ To a large extent
- ☐ To a very large extent
- ☐ *Don't know*
- ☐ *Not applicable*

3. The last time you required an urgent appointment with your GP, when did you receive the appointment?

- ☐ The same day
- ☐ The next day
- ☐ After 2 days
- ☐ After more than 2 days
- ☐ *Not applicable→ go to question 5*

4. Was this waiting time acceptable?

- ☐ Not at all
- ☐ To a small extent
- ☐ To some extent
- ☐ To a large extent
- ☐ To a very large extent

Questions 5 and 6 only apply to appointments that are not urgent (disregard appointments where urgent help is required).

5. How long do you usually have to wait for an appointment with your GP?

- ☐ 0-1 day
- ☐ 2-3 days
- ☐ 4-7 days
- ☐ 8-14 days
- ☐ More than two weeks
- ☐ *Not applicable → go to question 7*

6. Is this waiting time acceptable?

- ☐ Not at all
- ☐ To a small extent
- ☐ To some extent
- ☐ To a large extent
- ☐ To a very large extent

7. Do you normally see your own GP when you have an appointment?

- ☐ Yes
- ☐ No
- ☐ *Not applicable*

Assessment of GP

8. Do you think your GP takes you seriously?

- ☐ Not at all
- ☐ To a small extent
- ☐ To some extent
- ☐ To a large extent
- ☐ To a very large extent

9. Do you feel your GP spends enough time with you?

- ☐ Not at all
- ☐ To a small extent
- ☐ To some extent
- ☐ To a large extent
- ☐ To a very large extent

10. Do you feel your GP talks to you in a way you understand?

- ☐ Not at all
- ☐ To a small extent
- ☐ To some extent
- ☐ To a large extent
- ☐ To a very large extent

11. Do you feel your GP is professionally competent?

- ☐ Not at all
- ☐ To a small extent
- ☐ To some extent
- ☐ To a large extent
- ☐ To a very large extent

12. Do you feel your GP shows interest in your situation?

- ☐ Not at all
- ☐ To a small extent
- ☐ To some extent
- ☐ To a large extent
- ☐ To a very large extent

13. Do you feel your GP includes you as much as you would like in decisions concerning you?

- ☐ Not at all
- ☐ To a small extent
- ☐ To some extent
- ☐ To a large extent
- ☐ To a very large extent

14. Does your GP provide you with sufficient information about your health problems and treating them?

- ☐ Not at all
- ☐ To a small extent
- ☐ To some extent
- ☐ To a large extent
- ☐ To a very large extent

15. Does your GP provide you with sufficient information about the use and side effects of medication?

- ☐ Not at all
- ☐ To a small extent
- ☐ To some extent
- ☐ To a large extent
- ☐ To a very large extent
- ☐ *Not applicable*

16. Does your GP print out a list of your medicines when you change medication?

- ☐ Yes, always
- ☐ Yes, often
- ☐ Yes, sometimes
- ☐ No, rarely
- ☐ No, never
- ☐ *Not applicable*

17. During the past two years, have you been given the wrong medication or otherwise been incorrectly medicated by your GP?

- ☐ No, never
- ☐ Yes, once
- ☐ Yes, several times
- ☐ *Don't know*
- ☐ *Not applicable*

18. During the past two years, have you been incorrectly diagnosed by your GP?

- ☐ No, never
- ☐ Yes, once
- ☐ Yes, several times
- ☐ *Don't know*
- ☐ *Not applicable*

Organisation and other employees

19. Do you feel that your GP surgery is well organised?

- ☐ Not at all
- ☐ To a small extent
- ☐ To some extent
- ☐ To a large extent
- ☐ To a very large extent
- ☐ *Not applicable*

20. Do you feel the other employees are helpful and competent?

- ☐ Not at all
- ☐ To a small extent
- ☐ To some extent
- ☐ To a large extent
- ☐ To a very large extent
- ☐ *Not applicable*

21. Are you treated with courtesy and respect at reception?

- ☐ Not at all
- ☐ To a small extent
- ☐ To some extent
- ☐ To a large extent
- ☐ To a very large extent
- ☐ *Not applicable*

22. Do you generally have to wait in the waiting room beyond the agreed appointment time?

- ☐ Not at all
- ☐ To a small extent
- ☐ To some extent
- ☐ To a large extent
- ☐ To a very large extent
- ☐ *Not applicable*

23. Are conversations with your GP and other employees conducted in a way that protects your privacy?

- ☐ Not at all
- ☐ To a small extent
- ☐ To some extent
- ☐ To a large extent
- ☐ To a very large extent
- ☐ *Not applicable*

Coordination and cooperation

The GP is responsible for medical coordination and shall collaborate with other health services that the patient needs.

24. Do you feel that your GP effectively coordinates the range of health services available to you?

- ☐ Not at all
- ☐ To a small extent
- ☐ To some extent
- ☐ To a large extent
- ☐ To a very large extent
- ☐ *Don't know*
- ☐ *Not applicable*

25. Do you feel that your GP cooperates well with other services you need?

- ☐ Not at all
- ☐ To a small extent
- ☐ To some extent
- ☐ To a large extent
- ☐ To a very large extent
- ☐ *Don't know*
- ☐ *Not applicable*

26. Does your GP refer you for further investigation or to a specialist when you think you need it?

- ☐ Not at all
- ☐ To a small extent
- ☐ To some extent
- ☐ To a large extent
- ☐ To a very large extent
- ☐ *Not applicable*

Help to control and manage health problems

27. Does contact with your GP make you better able to understand your health problems?

- ☐ Not at all
- ☐ To a small extent
- ☐ To some extent
- ☐ To a large extent
- ☐ To a very large extent
- ☐ *Not applicable*

28. Does contact with your GP make you better able to take command of your health problems?

- ☐ Not at all
- ☐ To a small extent
- ☐ To some extent
- ☐ To a large extent
- ☐ To a very large extent
- ☐ *Not applicable*

29. Does contact with your GP make you better able to stay healthy?

- ☐ Not at all
- ☐ To a small extent
- ☐ To some extent
- ☐ To a large extent
- ☐ To a very large extent
- ☐ *Not applicable*

Internet/SMS

30. Check here if you would like your GP/GP surgery to offer any of the following services by internet or SMS.
You can choose several alternatives.

- ☐ Book an appointment
- ☐ Renew a prescription
- ☐ Access to your own medical information
- ☐ Access to your own children's medical information
- ☐ Video consultation
- ☐ Messaging services (e.g. via Pasientsky or Helsenorge.no)
- ☐ Other services by internet or SMS. What kind of services?

____

31. What services from your GP/GP surgery have you used by internet or SMS?
You can choose several alternatives.

- ☐ Booked an appointment
- ☐ Renewed a prescription
- ☐ Accessed my own medical information
- ☐ Accessed my own children`s medical information
- ☐ Video consultation
- ☐ Messaging services (e.g. via Pasientsky or Helsenorge.no)
- ☐ Other services by internet or SMS
- ☐ None of these

32. Are you satisfied with the availability of your GP surgery online and via SMS?

- ☐ Yes
- ☐ No
- ☐ *Don't know*

33. During the past two years, have you had a video consultation with your GP?

- ☐ No, never -> go to question 36
- ☐ Yes, once
- ☐ Yes, several times

34. Consider your most recent video consultation with your GP: were there any technical problems before or during the consultation?
You can choose several alternatives.

- ☐ Yes, with the internet connection
- ☐ Yes, with the sound
- ☐ Yes, with the picture
- ☐ Yes, other problems
- ☐ No, no problems

35. Overall, are you satisfied with your video consultations with your GP?

- ☐ Not at all
- ☐ To a small extent
- ☐ To some extent
- ☐ To a large extent
- ☐ To a very large extent

36. Is it important to you that your GP offers video consultations?

- ☐ Yes, but only during the COVID-19 pandemic
- ☐ Yes, both during and after the COVID-19 pandemic
- ☐ No

Coronavirus/COVID-19

37. During the past two years, has a test shown that you were infected with coronavirus?

- ☐ No, I have not been infected
- ☐ Yes, I have been infected
- ☐ *Don't know*

38. During the past two years, have you not booked an appointment with your GP/GP surgery even though you needed to?
You can choose several alternatives.

- ☐ No
- ☐ Yes, because I did not have time
- ☐ Yes, because I was concerned about being infected with the coronavirus
- ☐ Yes, because I was concerned about my GP's workload
- ☐ Yes, because I had symptoms of COVID-19 infection/respiratory infection
- ☐ Yes, because it was too difficult to book an appointment
- ☐ Yes, but for another reason
- ☐ I have not needed an appointment
- ☐ *Don't know*

39. During the past two years, have you been concerned that you would not get the health-care you need because your GP/GP surgery has been busy with the COVID-19 pandemic?

- ☐ Not at all
- ☐ To a small extent
- ☐ To some extent
- ☐ To a large extent
- ☐ To a very large extent
- ☐ *Don't know*
- ☐ *Not applicable*

Satisfaction with GP

40. All in all, are you satisfied with your GP?

- ☐ Not at all
- ☐ To a small extent
- ☐ To some extent
- ☐ To a large extent
- ☐ To a very large extent

Background information

41. How would you describe your *physical* health?

- ☐ Very poor
- ☐ Quite poor
- ☐ Neither poor nor good
- ☐ Quite good
- ☐ Very good

42. How would you describe your *mental* health?

- ☐ Very poor
- ☐ Quite poor
- ☐ Neither poor nor good
- ☐ Quite good
- ☐ Very good

43. Do you have a long-lasting health problem?
("Long-lasting" refers to a health problem that has lasted for at least six months or a more recent health problem that you believe will become long-lasting.)

- ☐ Yes, one
- ☐ Yes, two
- ☐ Yes, three or more
- ☐ No

44. Do you have any of the following long-lasting health problems/conditions?

You can choose several alternatives.

- ☐ High blood pressure (hypertension)
- ☐ Heart disease, including myocardial infarction
- ☐ Diabetes
- ☐ Asthma, or chronic lung diseases such as chronic bronchitis, emphysema or COPD
- ☐ Depression, anxiety or other mental health problems
- ☐ Substance abuse problems
- ☐ Cancer
- ☐ Musculoskeletal ailments, including joint pain or arthritis
- ☐ Had a stroke (CVA)
- ☐ Neurological disease/condition (e.g., epilepsy or migraine)
- ☐ Alzheimer’s disease or other types of dementia
- ☐ Other long-lasting health problems/ conditions. You can write what here: __________________________________
- ☐ No long-lasting health problems/conditions

45. What is your highest level of education?

- ☐ Compulsory primary school (grades 1-10)
- ☐ Upper secondary school
- ☐ College/university (1-4 years)
- ☐ College/university (more than 4 years)

46. Where were you born?

- ☐ Norway
- ☐ In a Nordic country (other than Norway)
- ☐ Western Europe (other than a Nordic country)
- ☐ EU country in Eastern Europe
- ☐ Eastern Europe (not a country in the EU)
- ☐ Africa
- ☐ Asia (including Turkey)
- ☐ North America
- ☐ South America or Central America
- ☐ Oceania

47. Did anyone help you fill out this form?

- ☐ Yes
- ☐ No

48. Who have you considered in this form?

- ☐ My current GP
- ☐ A previous GP
- ☐ Another doctor at the GP surgery
- ☐ Other

Feel free to write more about your experiences with your GP/GP surgery here:

Thank you for taking the time to complete the survey!

FHI
Folkehelseinstituttet
